# Nanosized Particles Assembled by a Recombinant Virus Protein Are Able to Encapsulate Negatively Charged Molecules and Structured RNA

**DOI:** 10.3390/polym13060858

**Published:** 2021-03-11

**Authors:** Hemalatha Mani, Yi-Cheng Chen, Yen-Kai Chen, Wei-Lin Liu, Shih-Yen Lo, Shu-Hsuan Lin, Je-Wen Liou

**Affiliations:** 1 Institute of Medical Sciences, Tzu Chi University, Hualien 97004, Taiwan; 104325118@gms.tcu.edu.tw (H.M.); 8871146@gms.tcu.edu.tw (S.-H.L.); 2 Department of Medicine, MacKay Medical College, New Taipei City 25245, Taiwan; chen15@mmc.edu.tw; 3 Department of Biochemistry, School of Medicine, Tzu Chi University, Hualien 97004, Taiwan; 99328101@gms.tcu.edu.tw (Y.-K.C.); 98322104@gms.tcu.edu.tw (W.-L.L.); 4 Department of Laboratory Medicine and Biotechnology, Tzu Chi University, Hualien 97004, Taiwan; losylo@mail.tcu.edu.tw

**Keywords:** nucleocapsid-like particle, protein-based nanoparticle, hepatitis C virus, drug delivery, protein–RNA interaction, fluorescent methods

## Abstract

RNA-based molecules have recently become hot candidates to be developed into therapeutic agents. However, successful applications of RNA-based therapeutics might require suitable carriers to protect the RNA from enzymatic degradation by ubiquitous RNases in vivo. Because of their better biocompatibility and biodegradability, protein-based nanoparticles are considered to be alternatives to their synthetic polymer-based counterparts for drug delivery. Hepatitis C virus (HCV) core protein has been suggested to be able to self-assemble into nucleocapsid-like particles in vitro. In this study, the genomic RNA-binding domain of HCV core protein consisting of 116 amino acids (p116) was overexpressed with *E. coli* for investigation. The recombinant p116 was able to assemble into particles with an average diameter of approximately 27 nm, as visualized by electron microscopy and atomic force microscopy. Measurements with fluorescence spectroscopy, flow cytometry, and fluorescence quenching indicated that the p116-assembled nanoparticles were able to encapsulate small anionic molecules and structured RNA. This study demonstrates methods that exploit the self-assembly nature of a virus-derived protein for nanoparticle production. This study also suggests that the virus-derived protein-assembled particles could possibly be developed into potential carriers for anionic molecular drugs and structured RNA-based therapeutics.

## 1. Introduction

RNA-based molecules, including antisense RNAs, small interfering RNAs, messenger RNAs, and RNA aptamers, have emerged as promising potential therapeutic agents for the treatments of a variety of diseases [1,2]. RNAs are polymeric molecules comprised of different numbers, and combinations of four major ribonucleotides differ by their nucleobases. Many of the therapeutic nucleic acids have been approved for clinical uses or have been under development [2,3,4]. While the sequence of a particular RNA is important for molecular function, in many cases, the folding of the RNA into proper secondary and tertiary structures is also crucial [1]. RNA-based therapeutics enjoy many advantages over protein-based and small molecule drugs: RNA-based therapeutics can be designed to target a wide range of targets; the generation time and production cost of RNA-based drugs are relatively lower than those of their protein-based counterparts [3]. However, the major problem encountered in the applications of RNA-based therapeutics is the in vivo stability of RNAs, which in their naked forms are easily degraded by blood and tissue RNases [2,5]. To solve this problem, researchers have applied several strategies to protect the RNAs and increase their half-life in the body. These methods include the applications of organic or inorganic polymer-based nanoparticles [6,7,8], encapsulation by lipid bilayers [9], and the use of protein-based vehicles [10]. RNA molecules are highly anionic due to their phosphate content in their sugar–phosphate backbone. As a result, most RNA delivery carriers are positively charged, and the cationic charges of polymers are used to electrostatically condense the negatively charged RNA into the carriers [2,7,11].

Recently, because of their safety and biodegradability, natural biopolymer-based particles or micelles have attracted attention from research scientists for their development of drug-delivery carriers [12]. The most frequently applied natural biopolymers for nanoparticle productions are polysaccharides, peptides, and proteins [12]. Protein-based nanoparticles are emerging as versatile carriers to deliver molecules for therapeutic and diagnostic purposes and becoming a potential alternative to synthetic polymer-based nanoparticles [13,14,15]. Hepatitis C virus (HCV) is an enveloped, single-stranded positive-sense RNA virus. Its genomic RNA consists of an open reading frame, flanked by two highly structured untranslated regions (UTRs) at its 5′ and 3′ ends [16]. The secondary and tertiary structures of the internal ribosome entry site (IRES) located in the 5′-UTR mediate the translation of virus polyproteins in host cells [16,17]. The core protein is one of the most conserved HCV proteins and is responsible for virus nucleocapsid assembly and packaging of the virus genomic RNA. The HCV core protein is highly basic, according to its amino acid sequence. However, its biochemical characteristics and structure are relatively poorly understood. The total length of the HCV core protein, consisting of 191 amino acids, can be divided into three domains [18]. Domain 1, with approximately 117 amino acids, is hydrophilic, containing a high proportion of basic amino acids, and is suggested to be involved in RNA-binding and nucleocapsid assembly [19]. This domain may form particles with only its N-terminal 75 amino acids [20,21]. Domain 2, from amino acids 118 to 174, is highly hydrophobic and is suggested to be involved in the targeting of HCV core protein to lipid droplets. The rest of the core protein is domain 3, a hydrophobic region functioning as the endoplasmic reticulum-anchoring domain [21]. A previous study has demonstrated that domain 1 alone can assemble into nucleocapsid-like particles even without interactions with virus genomic RNA in an *E. coli* expression system [22]. With the cationic nature of HCV core protein, it is possible that the self-assembled particles by HCV core protein can be applied as potential carriers for negatively charged molecules and structured RNA. This study aims to investigate the ability of the HCV core protein domain 1 (amino acids 1–116, p116) to encapsulate charged molecules and structured RNA into assembled particles by applying fluorescent techniques, including fluorescence spectroscopy, flow cytometry, and fluorescence quenching. This study also applied imaging techniques, such as electron microscopy and atomic force microscopy, to visualize nucleocapsid-like particle formation by the *E. coli* expressed recombinant virus protein. For the observation and detection, charged fluorescent dyes and fluorescent tRNA were used to represent charged molecules and structured RNA.

## 2. Materials and Methods

### 2.1. Overexpression of Recombinant HCV Core Proteins Using E. coli

The cDNA sequence of truncated HCV core protein 1–116 (domain 1, p116) was inserted into the pQE30 plasmid, a vector for expressing N-terminally His-tagged recombinant proteins (QIAGEN, Germantown, MD, USA), and transformed into *E. coli* cells (M15 strain, QIAGEN, Germantown, MD, USA). The bacteria were selected by Luria–Bertani (LB) broth (LAB M, Heywood, England) agar containing 50 μg/mL ampicillin (MDBio Inc., Rockville, MD, USA) and 10 μg/mL kanamycin (Progen, Australia). For core protein overexpression, transformed bacteria were cultured in 2xYT medium containing ampicillin and kanamycin (final concentrations of 50 μg/mL and 10 μg/mL, respectively) at 37 °C. 1 mM isopropyl-D-1-thiogalactopyranoside (IPTG, MDBio Inc., Rockville, MD, USA) was added to the bacteria culture for protein induction when the OD_595_ of the bacteria culture reached 0.5~0.6. After 3 h of induction, bacterial pellets were collected by centrifugation at 7000 rpm for 20 min.

### 2.2. Protein Purification and In Vitro Assembly of Nucleocapsid-Like Particles

For protein purification, bacteria pellets were resuspended in phosphate-buffered saline at 4 °C and lysed by French Press (Thermo Fisher Scientific, Waltham, MA, USA) with 2000 psi twice, then pelleted down with 13,000 rpm centrifugation for 30 min at 4 °C. The pellet was resuspended in urea buffer (phosphate buffer, pH 6.5, containing 8 M Urea, 500 mM NaCl, and 0.001% 2-mercaptoethanol) and centrifuged at 15,000 rpm, 4 °C for 20 min. Supernatants were collected and centrifuged at 20,000 rpm, 4 °C for 20 min. Supernatants were then applied to a His-tag affinity column (Ni-NTA, GE Healthcare Life Sciences, Uppsala, Sweden). The HCV core protein p116 was eluted by urea buffer containing 200 mM imidazole. The eluted fraction was pooled and further purified using a size-exclusion column (Sephacryl S-200 HR, GE Healthcare Life Sciences, Uppsala, Sweden) with a flow rate of 0.5 mL/min. The purity of purified p116 was verified by SDS–PAGE (Supplementary Figure S1). For in vitro assembly of nucleocapsid-like particles, 15 μM of purified p116 in urea buffer was dialyzed against refolding buffer (150 mM NaCl and 20 mM Na_2_HPO_4_, pH 6.5) three times for 3 h each at 4 °C with a 3.5 kDa cutoff membrane (Thermo Scientific, Waltham, MA, USA). The assembled particles were visualized using transmission electron microscopy (TEM) or atomic force microscopy (AFM).

### 2.3. Nucleocapsid-Like Particles Assembled by P116 with Fluorescent Molecules

**Figure 1:**
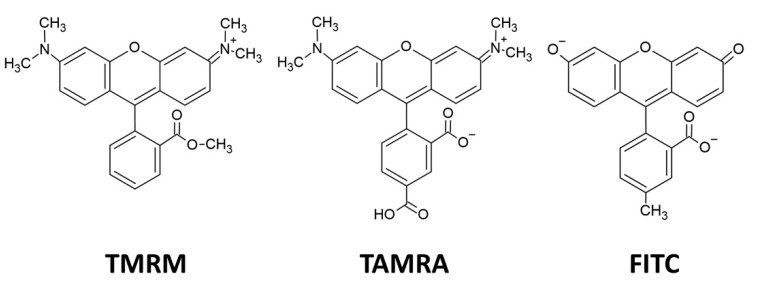
Structures of charged fluorescent molecules used in this study. Tetramethylrhodamine methyl ester (TMRM) has a net charge of +1, 5-carboxytetramethylrhodamine (TAMRA) has a net charge of 0, and fluorescein (FITC) has a net charge of −2.

In this study, for the investigation into whether the cationic p116 can specifically interact with negatively charged small molecules, three structurally similar fluorescent molecules with different net charges were used (Figure 1). Tetramethylrhodamine methyl ester (TMRM, Thermo Fisher Scientific, Waltham, MA, USA) is a positively charged molecule with an excitation wavelength at 549 nm and emission at 573 nm. 5-Carboxytetramethylrhodamine (TAMRA, Thermo Fisher Scientific, Waltham, MA, USA) is a net neutrally charged molecule also with excitation wavelength at 549 nm and emission at 573 nm. Fluorescein (FITC, Thermo Fisher Scientific, Waltham, MA, USA) is a negatively charged molecule with an excitation wavelength at 495 nm and emission at 513 nm. The fluorescent molecule was mixed with p116 before particle assembly in dialysis against refolding buffer. All fluorescence measurements were performed in a 1 cm quartz cuvette with a spectrofluorometer (FP-6500, Jasco, Tokyo, Japan).

### 2.4. Nucleocapsid-Like Particles Assembled by P116 with tRNA

Yeast tRNA powder (Sigma-Aldrich, St. Louis, MO, USA) was resolved in sterile water containing 0.1% (*v/v*) diethylpyrocarbonate and covalently labeled with fluorescein using a Mirus^TM^ Label IT nucleic acid labeling kit (Mirus, Madison, WI, USA). The fluorescence-labeled tRNA was mixed with p116 in urea buffer at a protein/tRNA molar ratio of 2/1. The mixture was then dialyzed against refolding buffer for particle assembly, as stated previously. The fluorescence of assembled particles was measured by using a flow cytometer (FACSCalibur, BD Biosciences, San Jose, CA, USA) equipped with an air-cooled 488 nm argon laser.

### 2.5. Fluorescence Quenching of Small Fluorescent Molecules and Fluorescent-Labeled tRNA

To determine whether the fluorescent charged molecules or fluorescent-labeled tRNA were indeed encapsulated inside the p116-assembled particles, fluorescence quenching experiments were performed. The fluorescence quenching experiments followed the methods described by Massou et al. [23] and Watt et al. [24], in which iodide (potassium iodide) was used as the quencher. The concentrations of quencher used in this study are indicated in the result figures.

### 2.6. Electron Microscopy and Atomic Force Microscopy

The morphology of p116-assembled particles was visualized using a transmission electron microscope (H-7500, Hitachi, Tokyo, Japan) with an accelerating voltage of 100 KeV. 10 μL of the p116-assembled particle-containing solution was placed onto a carbon-coated 300-mesh copper grid (PELCO, Ted Pella, Inc., Redding, CA, USA) and stayed for 1 min. The excess solution was wicked dry, and the sample was stained with 10 μL 1% phosphotungstic acid for 30 s (negative staining). The excess solution was again wicked dry, and the grid was allowed to air dry before TEM imaging. For AFM imaging, 50 μL the sample containing p116-assembled particles was dropped onto a freshly cleaved mica sheet and allowed to stay for 40 min for adsorption. The sheet was washed twice with Milli-Q water and air-dried for 48 h in the dry cabinet before AFM imaging. AFM measurements were performed with an atomic force microscope (NanoWizard^TM^, JPK Instruments, Berlin, Germany). The AFM probe used was 200 mm-long gold-coated cantilevers with oxide sharpened Si_3_N_4_ tips (Olympus, Tokyo, Japan). The spring constant for the cantilevers was 0.02 nN/nm. The scan rate was 1 Hz.

## 3. Results

### 3.1. Fluorescence Spectroscopy and Fluorescence Quenching of p116-Assembled Particles Interacting with Charged Small Molecules

**Figure 2:**
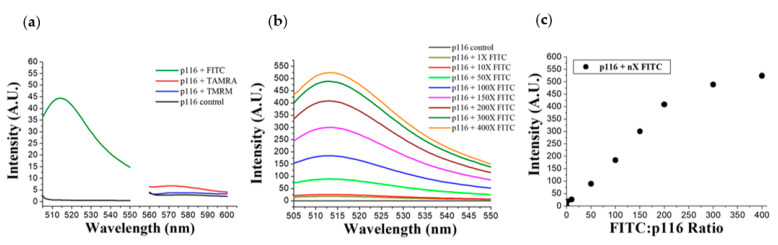
Fluorescence emission spectra of particles assembled by the RNA-binding domain 1 of HCV core protein (p116) in the presence of charged molecules. (**a**) Fluorescence spectra of p116-particles assembled in the presence of FITC, TAMRA, and TMRM; (**b**) fluorescence spectra of p116 interacted with different amounts of FITC. In (**a**,**b**), p116 control is the particles formed by p116 alone; (**c**) the fluorescence intensity at 513 nm of p116-assembled particles interacted with different molar ratios of FITC.

Figure 2a shows the fluorescence spectra of particles assembled by p116 in the presence of 2 × 10^−5^ M of negatively charged FITC, net neutrally charged TAMRA, or positively charged TMRM. As can be observed from Figure 2a, a significant increase in particle fluorescence was seen in the sample of p116 that interacted with the fluorescent dye FITC. The only very small increase in fluorescence was measured with the sample of p116 mixed with TAMRA. The increase in particle fluorescence of p116/TMRM mixed samples was measured and found to be not significant. These results indicated that the cationic p116 could specifically interact with negatively charged molecules. Figure 2b shows the fluorescence emission spectra of particles of p116 mixed with different molar ratios of FITC during assembly, and the fluorescence intensity at 513 nm for particles assembled with different FITC/p116 molar ratios are shown in Figure 2c. Fluorescence of particles was measured to be increased with an increased dose of the FITC dye mixed with p116 during particle assembly. The maximum fluorescence of particles measured was measured to be in the sample of FITC/p116 molar ratio of approximately 400.

### 3.2. Morphology of Nucleocapsid-Like Particle and Fluorescence Quenching of the Negatively Charged Dye

**Figure 3:**
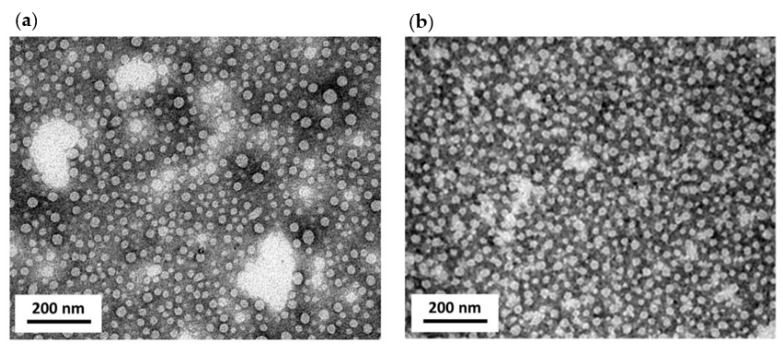
Transmission electron micrographs of nanoparticles assembled by p116. (**a**) a TEM image of particles assembled by p116 alone. (**b**) a TEM image of particles assembled by p116 added with the fluorescent molecule FITC during the particle formation process. The TEM imaging was conducted with electrons with an energy of 100 KeV. The scale bar in each image represents 200 nm. According to TEM imaging, the protein is able to assemble into nanosized particles with or without interacting with the anionic molecules.

**Figure 4:**
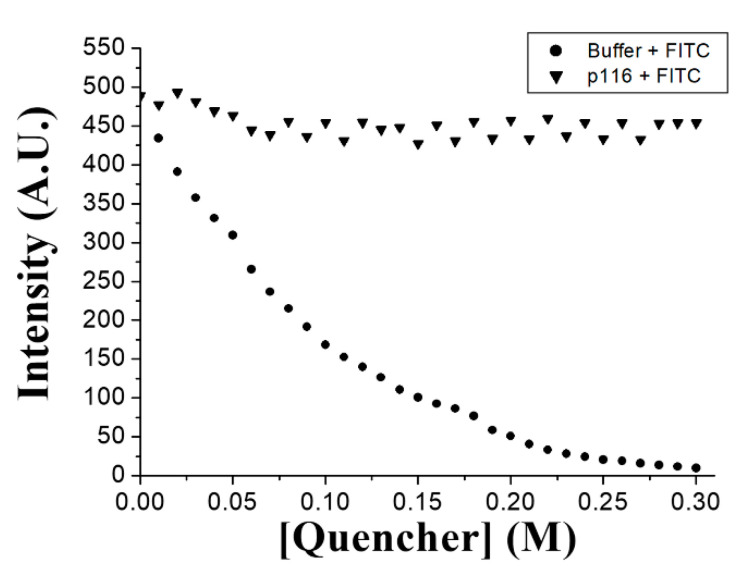
FITC fluorescence quenched by the quencher iodide. In the graphs, (▼) indicates the relative fluorescence intensity of free-form FITC in the presence of different quencher concentrations; (●) indicates the relative fluorescence intensity of FITC associated with p116-assembled particles in the presence of different quencher concentrations.

The morphology of the particles assembled by p116 was visualized with TEM. The TEM images of the samples containing p116-particles with or without interaction with FITC are shown in Figure 3. According to the electron micrographs, the particles with or without interaction to the negatively charged small molecules were similar in size and shape. No obvious difference was observed. The average diameters of particles assembled by p116 alone and p116 interacted with FITC were measured to be 26.4 nm and 26.6 nm, respectively. As indicated in the fluorescence measurements, the association with FITC dye did greatly increase the particle fluorescence. To rule out the possibility that the dye may just attach to the surface rather than be encapsulated into the particles, a fluorescence quencher was applied. Iodide is one of the most widely used fluorescence quenchers and was also applied in our study. In addition to FITC, the quencher was also able to quench the fluorescence of TMRM and TAMRA in their free forms (Supplementary Figure S2). However, as these two fluorescent dyes were found not to significantly associate with the p116-assembled particles, the fluorescence quenching experiments on particles assembled in the presence of TMRM and TAMRA were not performed. Figure 4 shows the fluorescence quenching of FITC with or without association to p116-particles by iodide. As can be seen, the quencher iodide was able to efficiently reduce the fluorescence of free-form FITC, and this reduction was in a dose-dependent manner. On the other hand, when FITC was associated with p116-assembled particles, the quenching effect caused by the quencher was greatly inhibited. More than 90% of the fluorescence emitted from the molecules was retained in fluorescence quenching experiments even when the concentration of quencher was increased to 300 mM. These results indicate that the quencher had difficulty in reaching the fluorescent dye FITC when the dye was associated with p116-assembled particles, meaning that the FITC dye was protected and encapsulated inside the particles.

### 3.3. p116-Assembled Particles Interacting with tRNA

**Figure 5:**
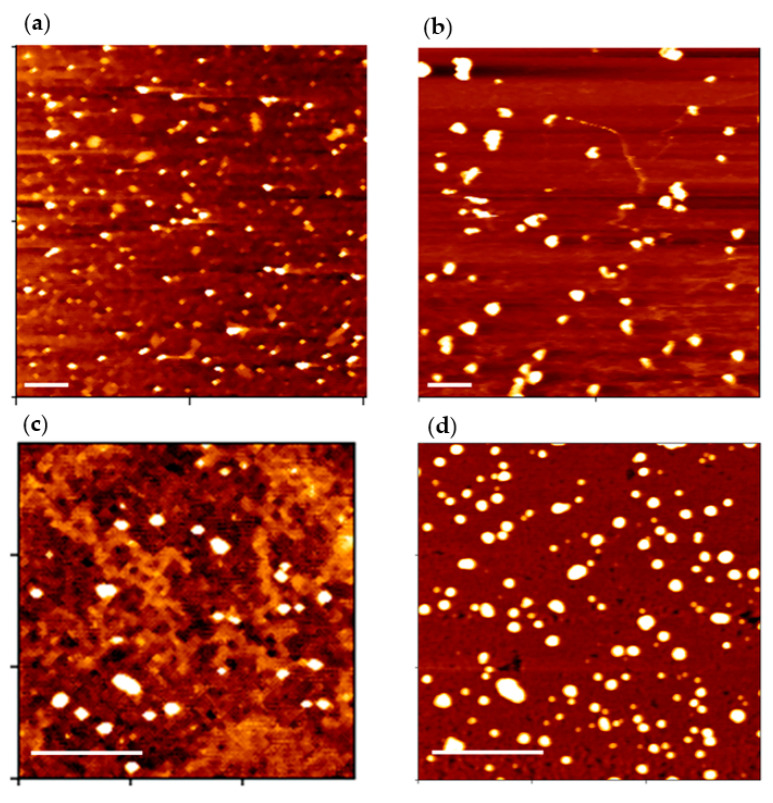
Atomic force microscopy (AFM) of the particles formed by p116 and p73. (**a**) An AFM image of particles assembled by p116 alone; (**b**) an AFM image of particles formed by p116 interacting with fluorescent tRNA during particle formation; (**c**) an AFM of particles assembled by p73 alone; (**d**) an AFM image of particles formed by p73 in the presence of fluorescent tRNA during the particle formation. In all these images, nucleocapsid-like particles were observed. The images shown are topographic images of AFM. Samples were imaged with a scan rate of 1 Hz and scan resolution of 512 × 512 pixels. The force applied by the AFM probe to the samples was 1 nN. The scale bar in each image represents 1 μm.

**Figure 6:**
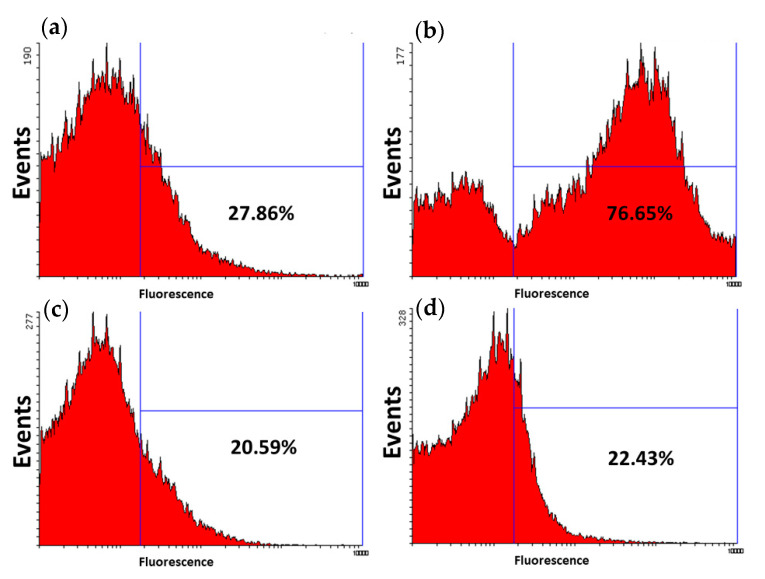
Fluorescence histograms of nucleocapsid-like particles in flow cytometry analysis. (**a**) Fluorescence distribution of the particles formed by p116 alone; (**b**) fluorescence distribution of particles formed by p116 with fluorescent-labeled tRNA; (**c**) fluorescence distribution of the particles formed by p73 alone; (**d**) fluorescence distribution of particles formed by p73 mixed with labeled tRNA.

Flow cytometry is a popular technique used to detect and measure physical and fluorescent characteristics of a population of cells, bacteria, or particles. In this study, the ability of p116-formed particles to associate and encapsulate structured RNA was investigated. We labeled the tRNA with fluorescent dye and used the fluorescent-labeled tRNA to interact with p116 during particle assembly. Flow cytometry was applied to measure the fluorescence of particles in populations. Before performing flow cytometry, we examined the particle forming ability of p116 when interacting with tRNA using AFM. Figure 5a is an AFM image of particles formed by p116 alone, while Figure 5b is an AFM image of particles assembled by p116 with tRNA. As indicated by AFM imaging, both p116 alone and p116 interacted with tRNA were able to form particles. As indicated in a previous study [21], the N-terminal 75 residues of the HCV core protein were sufficient to assemble and generate nucleocapsid-like particles in vitro; we also tried to use the truncated HCV domain 1 to interact with tRNA. In this study, recombinant truncated HCV core protein containing the N-terminal 73 amino acids (p73) was used. According to Figure 5c,d, p73 samples with or without the presence of tRNA were indeed able to form particles. We then used flow cytometry to measure the fluorescence of particles in the population for specific samples. The results of flow cytometry of p116- and p73-formed particles are shown in Figure 6. The interaction with tRNA indeed greatly increased the fluorescence of the p116-assembled particles measured. The particles with fluorescence level above the set threshold increased from approximately 28% in the sample of p116 alone (Figure 6a) to approximately 77% in the sample of p116 with tRNA (Figure 6b). However, it is very interesting to see that although p73 was able to form particles, the p73-formed particles seemingly tend not to associate with tRNA, at least not able to encapsulate the fluorescent-labeled tRNA in the particles. The addition of fluorescent tRNA to the p73 sample during particle formation did not increase the fluorescence of the particles (Figure 6d), as compared to the sample of p73 alone (Figure 6c).

**Figure 7:**
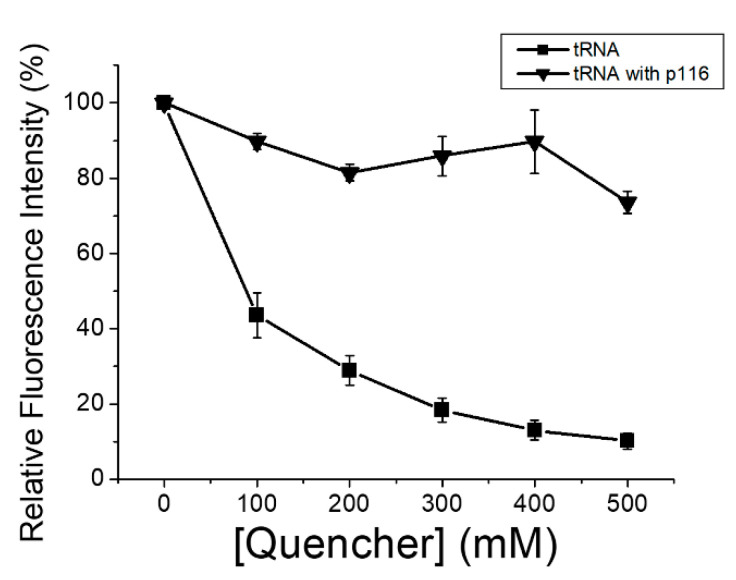
tRNA fluorescence quenched by the quencher iodide. In the graphs, (—■—) indicates the relative fluorescence intensity of free-form fluorescent-labeled tRNA in the presence of different quencher concentrations; (—▼—) indicates the relative fluorescence intensity of labeled tRNA associated with p116-assembled particles in the presence of different quencher concentrations. Each point is an average of three repeats; errors are standard deviations.

We also applied the fluorescence quenching technique to prove that the tRNA was indeed encapsulated and protected by the nucleocapsid-like particles formed by p116, and the results are shown in Figure 7. As clearly indicated in Figure 7, if not protected, the fluorescence of labeled tRNA was easily quenched by the quencher iodide. On the other hand, when the tRNA was associated with the p116-assembled particles, approximately 80% of the fluorescence can be retained when treated with the quencher at concentrations of as high as 400 mM. The fluorescence quenching experiments suggested that the tRNA was encapsulated in p116-formed particles, and the fluorescent probe on tRNA was protected by the particle-tRNA associations.

## 4. Discussion

Nanoparticles have been extensively investigated and applied as carriers for the delivery of chemicals and biomolecular drugs, as well as for the improvement of the half-life of the drugs by protecting the drugs from chemical or enzymatic degradation in the body [13,25]. Recently, natural materials, including proteins, are becoming attractive alternatives to synthetic polymers commonly used for nanoparticle productions. Protein-based nanoparticles have many advantages over synthetic polymer-based ones. Generally, they are easy to manipulate, often nontoxic, biocompatible, and biodegradable [13]. Various proteins, such as bovine/human serum albumin, fibroin, gelatin, gliadine, legumin, lipoprotein, keratin, sericin, collagen, and ferritin proteins, have currently been applied to generate protein nanoparticles [13,26]. Protein-based nanoparticles are commonly prepared through emulsion, electrospray, and desolvation methods [25]. In this study, we took advantage of the self-assembly nature of virus capsids for the generation of protein-based nanoparticles. HCV is an enveloped RNA virus whose core protein is the major component to directly interact with and encapsulate its genomic RNA. The HCV genomic RNA is highly structured [27]. The ability to encapsulate the genomic RNA suggests that the HCV core protein-assembled capsid might be used as a carrier for non-virus structured RNA molecules. The HCV core protein comprises three domains. While the hydrophobic domains 2 and 3 allow the protein to interact with host lipid droplets and to anchor the host endoplasmic reticulum, the highly cationic domain 1 is responsible for direct interaction to RNA. Previous studies have indicated that similar to those in some other viruses [28,29], the core protein of HCV can self-assemble into nucleocapsid-like particles in vitro [21,22,30,31,32], and this assembly does not necessarily require interaction with HCV genomic RNA. It is also suggested that the N-terminal 75 amino acids of the protein are required for particle assembly [21,33]. In this study, we over-expressed the RNA-binding domain 1 of HCV core protein (p116) using an *E. coli* expression system. The truncation of the hydrophobic tail of the HCV core protein can avoid undesired random aggregations of the protein due to hydrophobic interactions in solution and allow easier protein expression in bacterial cells. Only two liquid chromatography columns were required for protein purification, as His-tag was added to the recombinant p116 by the plasmid vector in the molecular cloning. As demonstrated in this study, the recombinant protein p116 was able to assemble into nucleocapsid-like particles. The average diameter of the particles was measured by electron microscopy to be approximately 27 nm, which is consistent with the measured sizes of self-assembled HCV nucleocapsid-like particles in other studies [21,22]. The sizes of the particles are also very close to unenveloped wild-type nucleocapsids of HCV [34,35], suggesting that the protein was properly folded. We also tested the particle formation ability of truncated core protein consisting of only the N-terminal 73 amino acids (p73). Our results showed that the p73 expressed in the *E. coli* system was able to form particles, and these results agreed with the finding in previous studies [21]. tRNA is a molecule that folds into secondary and tertiary structures. The possible interaction between HCV core protein and tRNA has been documented previously [22,30,32]; nevertheless, the end products resulted from the interaction have not yet been fully revealed. Here, by applying fluorescence techniques, we show that the tRNA was encapsulated into the particles assembled by p116, and the fluorescent-labeled tRNA was well protected by the nucleocapsid-like particles against the quench effects by iodide. We also found that the nucleocapsid-like particles assembled by p116 not only have the ability to encapsulate negatively charged tRNA but also be able to package negatively charged small molecules. Considering the finding in this study that the positively and net neutrally charged molecule could not associate with the particles, we strongly suspected that the interactions between the p116-assembled particles with structured RNA and small molecules largely depends on electrostatic interactions. The clusters of basic charged amino acids within the N-terminal 68 amino acids have been suggested to be critical for HCV capsid assembly [33]; these cationic clusters might also participate in the recruitment of negatively charged molecules. Both p116 and p73 contain these basic clusters, and both of the proteins were able to assemble into particles. However, it is very interesting to find that only p116-assembled particles were able to package fluorescent-labeled tRNA. Further investigations might be required to provide an answer to this difference.

RNA-based molecules represent a group of biomolecules exhibiting diverse biological functions and are under intensive research by academia and industry for their pharmaceutical and therapeutic potentials. However, successful applications of RNA-based molecules rely heavily on suitable carriers and delivery systems to overcome the problems caused by the ubiquitous RNases in the body. RNA molecules in nature spontaneously fold into specific secondary and tertiary structures [36,37] due to base-pairing, base-stacking, and base-backbone interactions [38]. Herein we report that it is possible to use self-assembled nanoparticles formed by a virus capsid protein-derived recombinant protein for encapsulation of structured non-virus RNA and small anionic molecules. The virus capsid-derived particles may be developed in the future into potential carriers and delivery systems for small molecular drugs and therapeutic RNA molecules.

## 5. Conclusions

In this study, we applied a bacterial overexpression system to produce a recombinant protein derived from the hepatitis C virus core protein. This recombinant viral protein was able to self-assemble into nucleocapsid-like particles as visualized by TEM and AFM. Fluorescence spectroscopy, flow cytometry, and fluorescence quenching experiments revealed that these nanosized particles were able to encapsulate fluorescent anionic small molecules and fluorescent-labeled structured RNA and to protect the fluorescence of molecules from the quenching effect by iodide. These nanoparticles self-assembled by the virus-derived protein can be used as a foundation for further development of carriers for the delivery of molecular or RNA-based therapeutics.

## Supplementary Materials

The following are available online at https://www.mdpi.com/2073-4360/13/6/858/s1, Figure S1: SDS–PAGE of p116 before and after purification using His-tag affinity and Sephacryl S-200 HR size-exclusion columns, Figure S2: Fluorescence quench of TMRM and TAMRA by iodide.
