# Supplementary material for: Nanosized Particles Assembled by a Recombinant Virus Protein Are Able to Encapsulate Negatively Charged Molecules and Structured RNA
Source: Polymers (Basel). 2021 Mar 11;13(6):858. doi: 10.3390/polym13060858 (PMC7998283; doi:10.3390/polym13060858)
Supplement: Supplementary file 1 [file polymers-13-00858-s001.pdf]

Supplementary Information

Nano-sized particles assembled by a recombinant virus protein are able to encapsulate negatively charged molecules and structured RNA

Hemalatha Mani [1], Yi-Cheng Chen [2], Yen-Kai Chen [3], Wei-Lin Liu [3], Shih-Yen Lo [4], Shu-Hsuan Lin [1] and Je-Wen Liou [1,3,*]

[1] Institute of Medical Sciences, Tzu Chi University, Hualien 97004, Taiwan
[2] Department of Medicine, MacKay Medical College, New Taipei City 25245, Taiwan
[3] Department of Biochemistry, School of Medicine, Tzu Chi University, Hualien 97004, Taiwan
[4] Department of Laboratory Medicine and Biotechnology, Tzu Chi University, Hualien 97004, Taiwan
[*] Correspondence: jwliou@mail.tcu.edu.tw

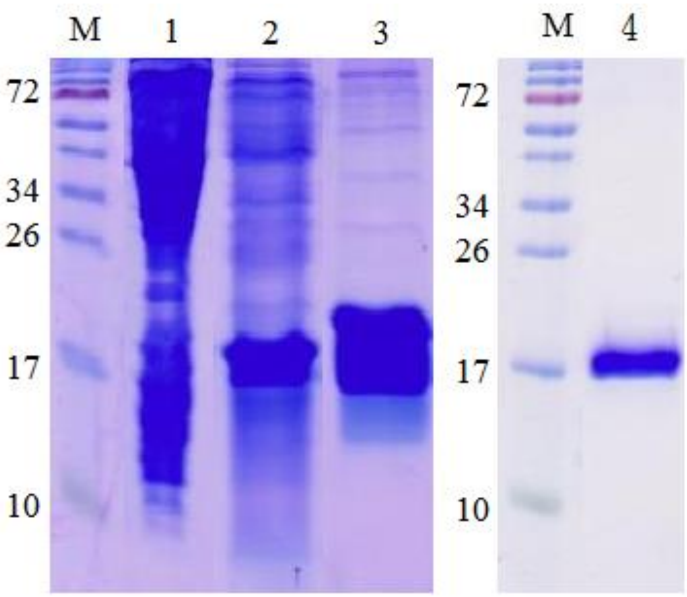

Figure S1. SDS–PAGE of p116 before and after purification using His-tag affinity and Sephacryl S-200 HR size-exclusion columns. lane 1: cell lysate; lane 2: total protein in 8M urea buffer; lane 3: p116 fraction after affinity column; lane 4: p116 fraction after size-exclusion column. After the purification, the purity is above 95%.

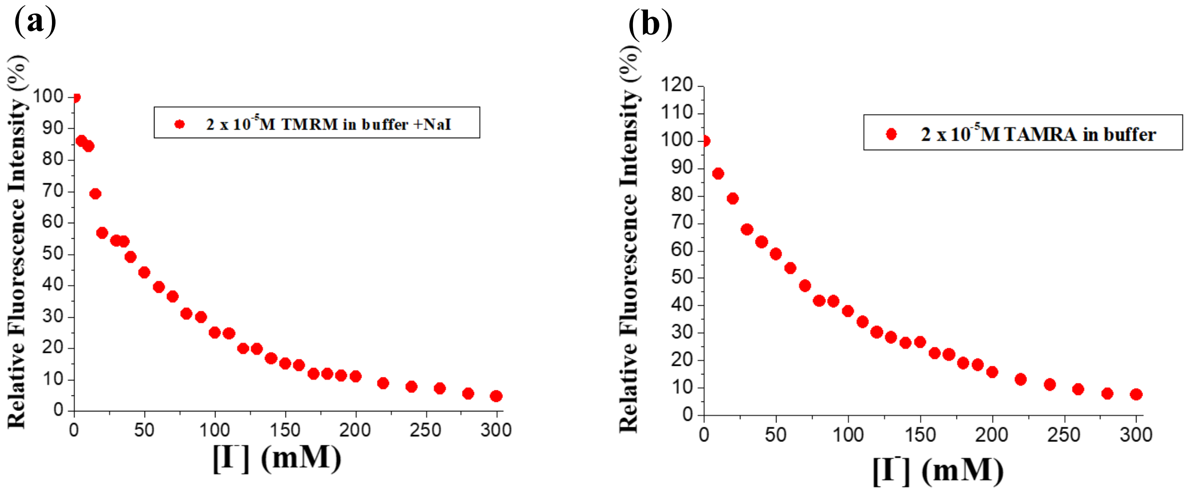

Figure S2. Fluorescence quench of TMRM and TAMRA by iodide. (**a**) The relative fluorescence intensity of positively charged TMRM in solution in presence of different concentrations of iodide; (**b**) The relative fluorescence intensity of net neutrally charged TARMA in solution in presence of different concentrations of iodide. As can be seen in the graphs, the relative fluorescence intensity of these two molecules decreased as the quencher concentration increased.
